# Rapid and reliable re-design of miniaturized microwave passives by means of concurrent parameter scaling and intermittent local tuning

**DOI:** 10.1038/s41598-023-34414-2

**Published:** 2023-05-05

**Authors:** Slawomir Koziel, Anna Pietrenko-Dabrowska

**Affiliations:** 1 Engineering Optimization and Modeling Center, Reykjavik University, 102 Reykjavik, Iceland; 2 Faculty of Electronics, Telecommunications and Informatics, Gdansk University of Technology, 80-233 Gdansk, Poland

**Keywords:** Electrical and electronic engineering, Computational science

## Abstract

Re-design of microwave passive components for the assumed operating frequencies or substrate parameters is an important yet a tedious process. It requires simultaneous tuning of relevant circuit variables, often over broad ranges thereof, to ensure satisfactory performance of the system. If the operating conditions at the available design are distant from the intended ones, local optimization is typically insufficient, whereas global search entails excessive computational expenses. The problem is aggravated for miniaturized components, typically featuring large numbers of geometry parameters. Furthermore, owing to their tightly-arranged layouts, compact structures exhibit considerable cross-coupling effects. In order to reliably evaluate electrical characteristics under such conditions full-wave electromagnetic (EM) analysis is mandatory. Needless to say, EM-driven design over broad ranges of operating frequencies is an arduous and costly endeavor. In this paper, we introduce a novel procedure for rapid and reliable re-design of microwave passives. Our methodology involves concurrent scaling of geometry parameters interleaved with local (gradient-based) tuning. The scaling stage allows for low-cost relocation of the operating frequencies of the circuit, whereas the optimization stage ensures continuous (iteration-wise) alignment of the performance figures with their target values. The presented framework is validated using several miniaturized microstrip couplers, re-designed over extended ranges of the center frequencies. For all considered structures, satisfactory designs are successfully identified despite the initial designs being distant from the targets, whereas local tuning turns out to be demonstrably inferior. Apart from its efficacy, one of the most important advantages of the proposed framework is its simplicity, and the lack of problem-dependent control parameters.

## Introduction

There is no doubt that optimization methods have now become an intrinsic part of the microwave design process. For many classes of circuits, rough initial designs can usually be obtained by means of the traditional, e.g., circuit theory methods (either in the form of explicit analytical formulas^1^, or equivalent networks^2^). Notwithstanding, conventional techniques and circuit models lack the accuracy whenever phenomena like dielectric and radiation losses or electromagnetic (EM) cross-coupling, have non-negligible effects on the system characteristics. Securing the best achievable performance requires cautious tuning of circuit dimensions^3^. Intricacy of modern microwave systems made experience-driven parametric studies virtually obsolete. Instead, rigorous numerical optimization is recommended^4^, which enables efficient handling of multiple variables, objectives, and constraints^5,6^. Unfortunately, optimization of microwave devices faces serious difficulties on its own. On the one hand, it is often necessary to handle large numbers of system variables, which is numerically demanding. On the other hand, accurate evaluation of circuits often involves CPU intensive full-wave EM analysis. Both factors contribute to considerable costs associated with parameter tuning^7^, as well as limited efficacy of conventional algorithms^8^. These problems are particularly pronounced for compact passive components constructed by meandering transmission lines (TL)^9–13^, compact microwave resonant cells (CMRCs)^14^, slow-wave phenomenon^15^, defected ground structures (DGS)^16,17^, electronic bandgap structures (EBG)^18,19^, or a variety of geometrical alterations (slots^20^, stubs^21^, shorting pins^22^, etc.). Miniaturization using the aforementioned approaches multiplies the number of degrees of freedom within the structures (e.g., a typical CMRC unit is described by at least four parameters versus two for a TL), which further exacerbates their design process, including optimization. In many cases, local algorithms^23^ have to be replaced by global procedures^24–29^, often leading to unmanageable computational expenses.

The literature provides a plethora of algorithmic solutions primarily aiming at the improvement of both the computational efficiency and reliability of the EM-driven optimization procedures. Acceleration methods include the incorporation of techniques for fast evaluation of circuit response gradients (adjoint sensitivities^30,31^, mesh deformation^32^, parallelization^33^), replacing numerical derivatives by updating formulas^34^, as well as utilization of sparse sensitivity updates^35–37^. In a more generic setting, surrogate-assisted procedures have been gaining considerable attention^38–41^, both in the context of physics-based (space mapping^42^, adaptive response scaling^43^, manifold mapping^44^), and data-driven models (radial basis functions^45^, kriging^46^, artificial neural networks^47^, ensemble learning^48^, support vector regression^49^), along with variable-resolution methods (co-kriging^50^, Bayesian model fusion^51^). Surrogate-based methods are applied for both local^52, 90–93^ and global optimization^53–55^, but also multi-criterial design^56–59^, and uncertainty quantification^60–62^. In terms of reliability improvements, in some cases combined with acceleration, some of the worth-mentioning techniques include efficient global optimizers (EGO)^63^, machine learning frameworks^64^, response feature technology^65^, cognition-driven design^66^, frequency-based regularization^67^, or adaptive design specification strategies^68^. Some of these approaches aim at facilitating exploration of the parameter space^63,69^, whereas others alleviate the difficulties pertinent to local routines (e.g., sensitivity to initial design^67,68^).

A particular type of an optimization task, which is of significant practical importance, is a re-design of a given microwave component to a different set of operating conditions (e.g., center frequency, power division ratio, etc.) or material parameters (e.g., substrate permittivity or thickness). The need for re-design may arise due to employing a circuit in a new application area, or implementing it on a different substrate. The associated numerical challenges are similar to those elaborated on at the beginning of this section: high computational cost, and potential reliability issues, especially for local search, or when the structure is to be scaled for operating parameters considerably misaligned with those at the current design. A number of frameworks have been developed to facilitate the dimension scaling process, including analytical design curves^70^, utilization of inverse surrogate models^71,72^, response features^73^, as well as more sophisticated frameworks that enable control of both the major operating conditions (e.g., center frequencies) and supplementary performance figures (e.g., power split ratios)^74^. The major drawback of the aforementioned techniques constitutes a high initial cost necessary to construct the surrogate models, which are typically rendered using pre-optimized reference designs^71^. Utilization of generic surrogate modeling techniques is another option^75–78^; however, setting up dependable metamodels over extended ranges of cicuit dimensions and material parameters (otherwise necessary to ensure design utility) is exacerbated by the curse of dimensionality and highly nonlinear microwave components characteristics. A possible workaround is a utilization of performance-driven modeling methods, which permit a rendition of accurate surrogates at a fraction of CPU costs incurred by conventional techniques^79–81^.

This article introduces a novel technique for fast and reliable re-design of passive microwave devices. Our methodology employs a concurrent scaling of geometry parameters, which is interleaved with local tuning. The role of the scaling stage is to perform large-scale design relocation of the circuit operating frequency at low computational cost. The tuning stage aims at improving the performance parameters of the circuit before launching the next scaling step. Eventually, i.e., upon relocating the operating frequency near the target, the process defaults to gradient-based optimization. The efficacy of the introduced technique is verified using several compact microstrip couplers, which are re-designed for center frequencies distant from those at the initial designs. Comparative experiments also indicate that straightforward local search normally fails under these conditions. Apart from its reliability, the proposed framework exhibits other attractive features, including simple implementation, easy handling, in particular, the lack of problem-dependent control parameters that need to be tuned, and low cost. The latter is related to the fact that our method does not utilize any surrogate models, and, consequently, there is no initial cost associated with the acquisition of either training data or pre-optimized reference designs.

## Circuit re-design using concurrent scaling and intermittent local tuning

This section explains the constituent parts of the introduced re-design methodology. We start by formulating the design problem in Sect. "Problem statement". Section "Concurrent parameter scaling" discusses the concurrent parameter scaling stage. Section "Intermittent local tuning" describes the local (gradient-based) tuning step, which is carried out to ensure sufficient performance of the circuit before executing the subsequent scaling step. The complete optimization procedure has been summarized in Sect. "Complete optimization procedure".

### Problem statement

In the following, ***x*** = [*x*_1_ … *x*_*n*_]^*T*^ represents the vector of design parameters of the considered circuit (i.e., its dimensions). We will also denote by ***S***(***x***) the EM-simulated scattering parameters. For individual *S*-parameters, we will use the symbol *S*_*jk*_(***x***,*f*) to denote the value of *S*_*jk*_ at the design ***x*** and frequency *f*. Finally, we denote by ***F***_*t*_ = [*F*_*t*.0_ *F*_*t*.1_ … *F*_*t.N*_]^*T*^ the target vector of operating parameters with the first entry, *F*_*t*.0_, being the operating frequency. The remaining entries denote the target values for other figures of interest, such as bandwidth, footprint area, power split ratio, material parameters of the substrate the circuit is to be implemented on, etc. At this point, it should be emphasized that the optimization technique introduced in this paper pertains to single-band structures. A generalization for multi-band systems will be discussed elsewhere.

The quality of design ***x*** with respect to the target vector ***F***_*t*_ is quantified using the objective function *U*(***x***,***F***_*t*_), which should be defined so that lower values of *U* correspond to better designs, i.e., those that meet the design specifications to the fuller extent. Having the objective function, the optimization task is formulated as

1 $${\boldsymbol{x}}^{*} = U^{*} ({\boldsymbol{F}}_{t} ) = \arg \mathop {\min }\limits_{{{\boldsymbol{x}} \in X}} U({\boldsymbol{x}},{\boldsymbol{F}}_{t} )$$

Here x^*^ is the optimum design to be identified. The optimum solution is sought for in the design space *X*, which is normally delimited using the lower bounds ***l*** = [*l*_1_ … *l*_*n*_]^*T*^ and the upper bound ***u*** = [*u*_1_ … *u*_*n*_]^*T*^ for the system parameters, i.e., *l*_*k*_ ≤ *x*_*k*_ ≤ *u*_*k*_, *k* = 1, …, *n*. In some cases, we also have additional constraints: inequality or equality ones. However, in the following, we assume for simplicity that the only geometrical constraints are those related to ***l*** and ***u***.

Table 1 gathers a few exemplary design tasks for microwave components. Note that in the presented formulations, some of the design objectives are treated as constraints, and handled using a penalty function approach^82^. For more information about penalty functions see, e.g.,^82,83^.

**Table 1:** Selected examples of design tasks for passive microwave components.

Task description	Target operating vector	Objective function^$^
Improve matching |*S*_11_| of impedance transformer over the frequency range *f*_*L*_ to *f*_*H*_	***F***_*t*_ = [*F*_*t*.0_ *F*_*t.*1_]^*T*^ where *F*_*t.*0_ = (*f*_*L*_ + *f*_*H*_)/2 – center frequency *F*_*t.*1_ = [*f*_*H*_ – *f*_*L*_] – bandwidth	$$U({\boldsymbol{x}},{\boldsymbol{F}}_{t} ) = \max \left\{ {F_{t.0} - \frac{{F_{t.1} }}{2} \le f \le F_{t.0} + \frac{{F_{t.1} }}{2}:\;|S_{11} ({\boldsymbol{x}},f)|} \right\}$$
Improve matching |*S*_11_| and isolation |*S*_41_| of a microwave coupler, and ensure power split *d*_*S*_(***x***,*f*) =| |*S*_21_(***x***,*f*)| – |*S*_31_(***x***,*f*)| |= *K*, both at the center frequency *f*_0_; the circuit is to be implemented on the substrate of permittivity ε_*r*_	***F***_*t*_ = [*F*_*t*.0_ *F*_*t.*1_ *F*_*t.*2_]^*T*^ where *F*_*t.*0_ = *f*_0_ – center frequency *F*_*t.*1_ = *K* – power split ratio *F*_*t.*2_ = ε_*r*_ – substrate permittivity	$$\begin{gathered} U({\boldsymbol{x}},{\boldsymbol{F}}_{t} ) = \max \left\{ {|S_{11} ({\boldsymbol{x}},F_{t.0} )|,|S_{41} ({\boldsymbol{x}},F_{t.0} )|} \right\} \\ + \beta c({\boldsymbol{x}},{\boldsymbol{F}}_{t} )^{2} \\ \end{gathered}$$ where $$c({\boldsymbol{x}},{\boldsymbol{F}}) = |S_{31} ({\boldsymbol{x}},F_{t.0} )| - |S_{21} ({\boldsymbol{x}},F_{t.0} )| - F_{t.1}$$
Reduce footprint *A*(***x***) of a microstrip coupler while maintaining matching and isolation at –20 dB or better, and equal power split ratio, both over the bandwidth [*f*_0_ – *B*/2, *f*_0_ + *B*/2]	***F***_*t*_ = [*F*_*t*.0_ *F*_*t.*1_ *F*_*t.*2_]^*T*^ where *F*_*t.*0_ = *f*_0_ – center frequency *F*_*t.*1_ = *B* – target bandwidth *F*_*t.*2_ = –20 dB – acceptance threshold for |*S*_11_| and |*S*_41_|	$$U({\boldsymbol{x}},{\boldsymbol{F}}_{t} ) = A({\boldsymbol{x}}) + \beta_{1} c_{1} ({\boldsymbol{x}},{\boldsymbol{x}}_{t} )^{2} + \beta_{2} c_{2} ({\boldsymbol{x}},{\boldsymbol{F}}_{t} )^{2}$$ where $$c_{1} ({\boldsymbol{x}},{\boldsymbol{F}}_{t} ) = \max \left\{ \begin{gathered} F_{t.0} - \frac{{F_{t.1} }}{2} \le f \le F_{t.0} + \frac{{F_{t.1} }}{2}: \\ \max \left\{ {\frac{{\max \left\{ {|S_{11} ({\boldsymbol{x}},f)|,|S_{41} ({\boldsymbol{x}},f)|} \right\} + F_{t.2} }}{{F_{t.2} }}} \right\} \\ \end{gathered} \right\}$$ $$c_{2} ({\boldsymbol{x}},{\boldsymbol{F}}_{t} ) = \max \left\{ \begin{gathered} F_{t.0} - \frac{{F_{t,1} }}{2} \le f \le F_{t.0} + \frac{{F_{t,1} }}{2}: \hfill \\ \left| {|S_{31} ({\boldsymbol{x}},f)| - |S_{21} ({\boldsymbol{x}},f)|} \right| \hfill \\ \end{gathered} \right\}$$

^$^The coefficient *β* > 0 is a penalty factor that controls the contribution of the penalty terms to the merit function^82^.

### Concurrent parameter scaling

The physical size of conventional (transmission-line-based) microstrip components affects the guided wavelength^1, 2^. Consequently, approximate re-design of circuit parameters, aimed at relocating the operating frequency of the system, can be achieved through a concurrent dimension scaling, i.e., jointly increasing or decreasing the geometry parameters. In this work, we describe this process as a transformation *M*_*S*_(***x***,*α*), defined as

2 $$M_{S} ({\boldsymbol{x}},\alpha ) = M_{S} ([x_{1} \;...\;x_{n} ]^{T} ,\alpha ) = \left[ {\min (u_{1} ,\max (l_{1} ,\alpha x_{1} ))\;\;...\;\;\min (u_{n} ,\max (l_{n} ,\alpha x_{n} ))} \right]^{T}$$

Recall that ***l*** = [*l*_1_ … *l*_*n*_]^*T*^ and ***u*** = [*u*_1_ … *u*_*n*_]^*T*^ stand for the lower and upper bounds for geometry parameters. The transformation *M*_*S*_ is defined to ensure that the scaling process leaves the design within the assumed design space *X*.

**Figure 1:**
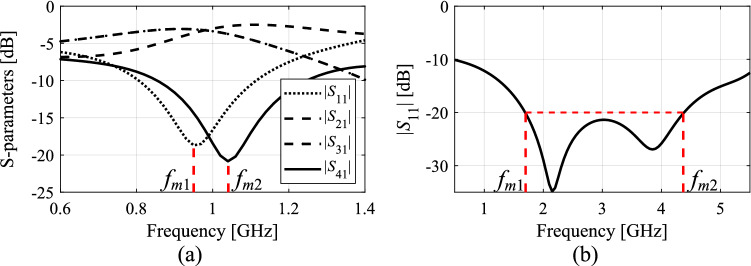
Extracting approximate center frequency *f*_*a*.0_ of a circuit: (**a**) microwave coupler: *f*_*a*.0_ = (*f*_*m*1_ + *f*_*m*2_)/2, where *f*_*m*1_ and *f*_*m*2_ are the frequencies of the minima of |*S*_11_| and |*S*_41_|, respectively; (**b**) impedance matching transformer: *f*_*a*.0_ = (*f*_*m*1_ + *f*_*m*2_)/2, where *f*_*m*1_ and *f*_*m*2_ are the frequencies corresponding to the left- and right-hand-side edges of the –20 dB bandwidth of the circuit, respectively.

Let *f*_*a.*0_(***x***) be the actual center frequency of the circuit at hand at a certain design ***x***. It can be extracted from the EM-simulated system responses, and defined depending on the particular type of a circuit. Typically, it would be the arithmetic average of the frequencies corresponding to the specific features of the system responses, e.g., the minima of the matching and isolation response for a coupler (cf. Figure 1a), or the frequencies determining the bandwidth for a broadband impedance transformer (cf. Figure 1b).

**Figure 2:**
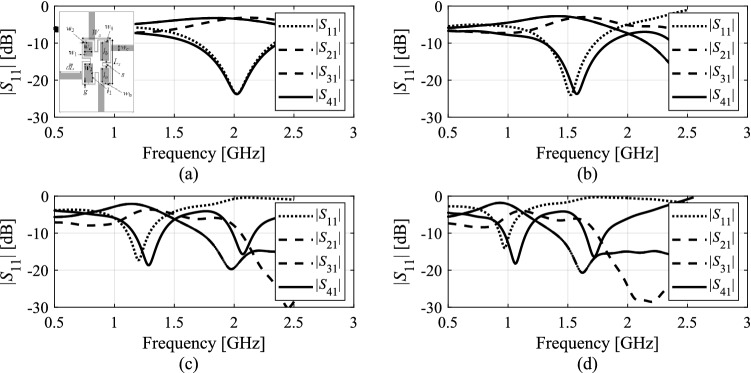
Concurrent dimension scaling of a compact branch-line coupler. EM-simulated *S*-parameters at: (**a**) initial design ***x***, (**b**) design obtained as *M*_*S*_(***x***,1.2), (**c**) design obtained as *M*_*S*_(***x***,1.4), (**d**) design obtained as *M*_*S*_(***x***,1.6). Note increasing distortion of electrical characteristics, especially for *α* > 1.4.

Here, the concurrent dimension scaling is executed to re-align the operating frequency of the circuit with its target value *F*_*t*.0_. The scaling coefficient *α* is computed as

3 $$\alpha = \min \left\{ {\alpha_{\max } ,\max \left\{ {\alpha_{\min } ,\frac{{f_{a.0} ({\boldsymbol{x}})}}{{F_{t.0} }}} \right\}} \right\}$$

where *α*_min_ and *α*_max_ are user-defined lower and upper bounds. These are introduced to avoid excessive scaling, which may be detrimental to the shape of the circuit characteristics. This is particularly important for compact structures, where interrelations between geometry parameters and electrical responses are rather complex. Concurrent scaling usually leads to response distortion if carried out using *α* which is significantly different from the unity. Figure 2 illustrates this for an exemplary microstrip coupler scaled using *α* = 1.2, 1.4, and 1.6. Typically, we would set *α*_min_ = 0.7, and *α*_max_ = 1/*α*_min_, to be on a safe side.

Concurrent scaling is followed by the extraction of the center frequency *f*_*a.*0_. If the extraction is impossible due to heavy distortion of the circuit characteristics, the scaling is repeated with an updated coefficient *α*/*m*_*α*_ (for *α* > 1) or *m*_*α*_*α* (for *α* < 1), where *m*_*α*_ is a control parameter, normally set to *α*_max_^1/2^. If repeated scaling still fails, it is abandoned altogether in a current iteration of the optimization process, and the design is improved using local tuning (cf. Sect. "Intermittent local tuning") before attempting the scaling process again.

### Intermittent local tuning

Local tuning is executed after applying the concurrent dimension scaling of Sect. "Concurrent parameter scaling". Its role is to improve the quality of the current design before launching the scaling again, or to finalize the optimization process if the current operating frequency *f*_*a*.0_ is sufficiently close to the target *F*_*t*.0_. The tuning is realized as the iterative gradient-based algorithm with numerical derivatives^84^.

Given the design ***x***^(*i*)^ obtained using the scaling procedure (or the previous application of the tuning routine), the new candidate design ***x***^(*i*+1)^ is rendered as

4 $${\boldsymbol{x}}^{(i + 1)} = \arg \mathop {\min }\limits_{\substack{ {\boldsymbol{x}} \in X \\ ||{\boldsymbol{x}} - {\boldsymbol{x}}^{(i)} || < d^{(i)} } } U_{L} ({\boldsymbol{x}},{\boldsymbol{F}}_{a} )$$

where the objective function *U*_*L*_ is defined similarly as the original function *U* (cf. Sect. "Problem statement"); yet, it is calculated using the linear expansion model of the circuit characteristics

5 $${\boldsymbol{L}}^{(i)} ({\boldsymbol{x}}) = {\boldsymbol{S}}({\boldsymbol{x}}^{(i)} ) + {\boldsymbol{J}}_{S} ({\boldsymbol{x}}^{(i)} ) \cdot ({\boldsymbol{x}} - {\boldsymbol{x}}^{(i)} )$$

The Jacobian matrix ***J***_*S*_ is evaluated using finite differentiation^85^. The vector ***F***_*a*_ is the current target objective vector, defined as

6 $${\boldsymbol{F}}_{a} = \left[ {f_{a.0} \;F_{t.1} \;...\;F_{t.N} } \right]^{T}$$

i.e., it coincides with ***F***_*t*_ except the first entry, which is replaced by the existent operating frequency of the circuit extracted at the design ***x***^(*i*)^. As mentioned before, the tuning step (4), (5) aims at improving the design quality in terms of its performance parameters with respect to the current operating parameters, and prior to executing another round of concurrent scaling.

The size *d*^(*i*)^ of the search region is adaptively adjusted contingent upon the gain ratio *r* = [*U*(***x***^(*i*+1)^,***F***_*a*_) – *U*(***x***^(*i*)^,***F***_*a*_)]/[*U*_*L*_(***x***^(*i*+1)^,***F***_*a*_) – *U*_*L*_(***x***^(*i*)^,***F***_*a*_)] (i.e., increased if *r* is close to 1, and decreased if it is close to zero^84^). Also, the tried design is retained if *r* > 0, i.e., the improvement of the original objective function *U* (computed using EM simulation data) has been observed.

If *f*_*a.*0_ is sufficiently close to *F*_*t*.0_, concurrent scaling is no longer executed, and the tuning process (4), (5) is continued until convergence. Here, the termination condition is ||***x***^(*i*+1)^ – ***x***^(*i*)^||< *ε*_*x*_ (when converging in argument), or *d*^(*i*)^ < *ε*_*x*_ (search region reduction); we set *ε*_*x*_ = 10^–3^.

### Complete optimization procedure

This section puts together the entire optimization procedure involving the concurrent scaling of Sect. "Concurrent parameter scaling", and local tuning of Sect. "Intermittent local tuning". The major assumption here is that the initial design ***x***^(0)^ is of sufficient quality to allow successful scaling, i.e., so that the scaled circuit has identifiable operating frequency (cf. Sect. "Concurrent parameter scaling"). This assumption normally holds, because the purpose of the described procedure is circuit re-design. Otherwise, the execution of the procedure should be preceded by local optimization with respect to the current (actual) operational frequency of the circuit.

Table 2 gathers the control parameters of the presented algorithm. It should be noted that we only have three independent parameters *α*_min_, *ε*_*x*_, and *dF*_0_. None of these is critical. For example, setting *α*_min_ sufficiently close to unity is a safer option (to avoid abrupt design relocations), and the performance of the optimization process will be more or less invariant of the choice. The second parameter decides upon the resolution of the optimization process, whereas the last one can be set as a small fraction (e.g., five percent) of *F*_*t*.0_. As a matter of fact, setting *dF*_0_ to anything less than half of the expected circuit bandwidth is normally sufficient to ensure adequate operation of the procedure.

**Table 2:** Proposed optimization procedure: Control parameters.

Parameter	Recommended value	Comments
*α*_min_	0.7	Minimum value of the scaling coefficient *α* for concurrent scaling (cf. Sect. "Concurrent parameter scaling")
*α*_max_	1/*α*_min_	Maximum value of the scaling coefficient *α* for concurrent scaling (cf. Sect. "Concurrent parameter scaling")
*m*_*α*_	*α*_max_^1/2^	Updating factor for the scaling coefficient *α*
*ε*_*x*_	10^–3^	Termination threshold (cf. Sec. "Intermittent local tuning")
*dF*_0_	0.05⋅*F*_*t.*0_	Concurrent scaling threshold (scaling enabled if |*f*_*a.*0_ – *F*_*t*.0_|> *dF*_0_)

**Figure 3:**
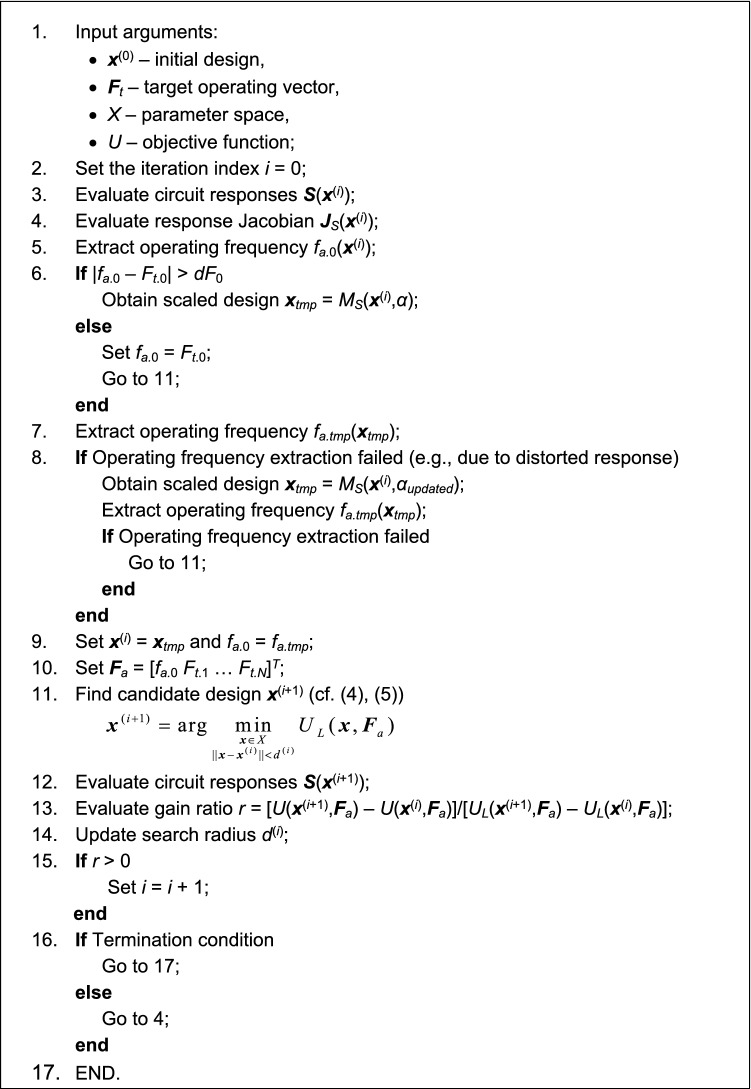
Pseudocode of the proposed re-design algorithm with concurrent parameter scaling and intermittent local tuning.

Figure 3 presents the pseudocode of the re-design procedure. It can be noted that the concurrent scaling is only performed (Step 6) if the actual center frequency is too far from to the target, i.e., if |*f*_*a.*0_ – *F*_*t.*0_|> *dF*_0_. If the scaling is unsuccessful, that is, the operating frequency cannon be extracted at the candidate design, it is repeated with the updated scaling coefficient *α*_*update*_ (Step 8).

**Figure 4:**
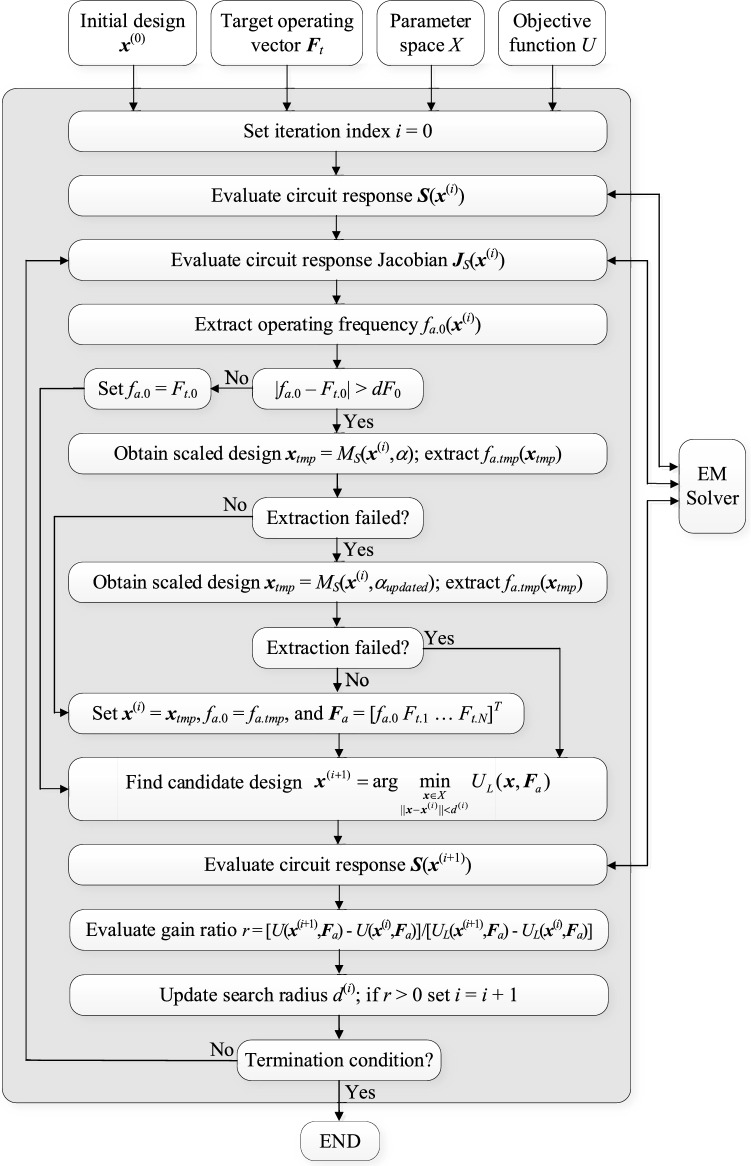
Flow diagram of the proposed re-design algorithm with concurrent parameter scaling and intermittent local tuning.

The next stage is local tuning (Step 11), which aims at improving the design quality in terms of the objective function value. At this stage, the circuit is optimized using the objective function *U* computed for the current center frequency *f*_*a*.0_. Local tuning is performed intermittently, following subsequent rounds of concurrent scaling. If the operating frequency of the circuit becomes sufficiently close to the target, concurrent scaling is no longer executed, and the final design is produced through local optimization. For additional explanation, Fig. 4 provides a flow diagram of the proposed algorithm.

## Verification case studies

This section summarizes the results of the numerical experiments conducted to demonstrate the operation and performance of the introduced optimization technique. The experiments were designed to verify the capability of our procedure to handle circuit re-design over wide range of operating frequencies, with the initial design corresponding to the center frequency significantly different from the target one. Under such conditions, conventional local tuning normally fails, which was also corroborated by carrying out the appropriate optimization runs. A remark should be made that all of the benchmark microwave components have been already experimentally validated (both in the source papers^86–89^, as well as in our previous work, e.g.,^81^). Thus, the experimental validation of the optimized designs has not been provided, as being immaterial to the scope of the paper.

### Verification circuits

**Figure 5:**
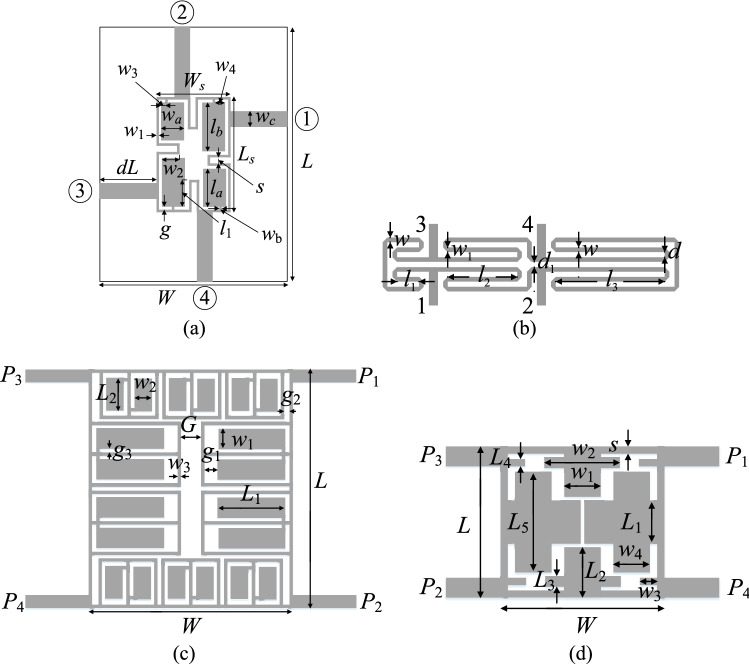
Microstrip couplers employed as verification case studies to validate the re-design procedure proposed in this work: (**a**) Circuit I: miniaturized branch-line coupler (BLC)^86^, (**b**) Circuit II: rat-race coupler of transmission lines folded^87^, (**c**) Circuit III: BLC with microstrip cells^88^, (**d**) Circuit IV: compact BLC (non-equal power split)^89^.

**Figure 6:**
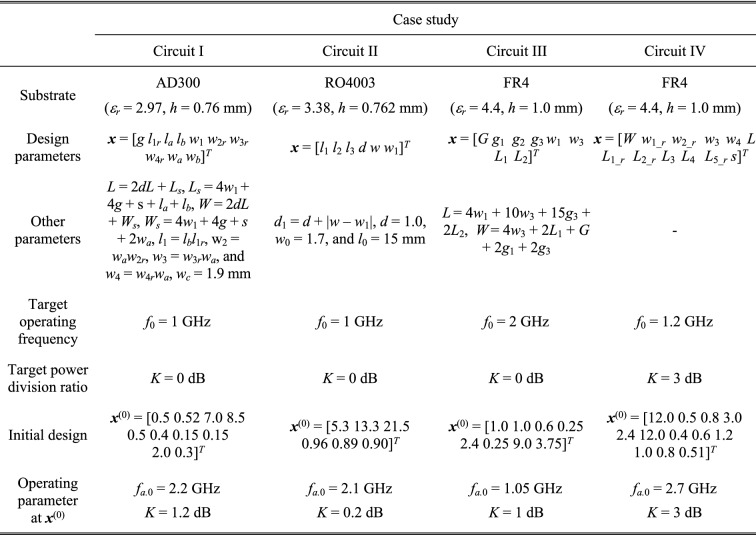
Microwave couplers used for validating of the proposed re-design procedure.

Verification experiments have been performed using four compact microstrip couplers shown in Fig. 5. The computational models are implemented and simulated using the time-domain solver of CST Microwave Studio. The important data concerning the structures in Fig. 5 (material parameters of the substrate, independent and dependent geometry parameters, target operating frequencies, initial designs) have been shown in Fig. 6. For all verification structures, we aim at re-designing the circuit from the given initial design to the center frequency listed in the table as *F*_*t.*0_. The design task is formulated as in Table 1 (second row). For Circuits I through III, the target power split ratio is 0 dB, whereas for Circuit IV, it is 3 dB. The target operating vector is ***F***_*t*_ = [*F*_*t*.0_ *F*_*t.*1_ *F*_*t.*2_]^*T*^, where *F*_*t.*0_ = *f*_0_ is the center frequency, *F*_*t.*1_ = *K* is the target power split ratio, whereas *F*_*t.*2_ = ε_*r*_ is the substrate permittivity.

Observe that the proposed optimization method is a general one and may be successfully applied for design optimization of verification structures other than those presented in Fig. 5, such as, e.g., antenna structures. The sole limitation of the introduced optimization technique is that it is capable of handling single-band structures. A generalization for multi-band systems will be a focus of the future work.

### Numerical results and discussion

The procedure of Sect. "Circuit re-design using concurrent scaling and intermittent local tuning" along with the conventional gradient-based search^84^ have been applied to optimization of Circuits I, II, III, and IV. In all cases, local tuning starting from the initial designs listed in Table 3 failed, i.e., the algorithm was unable to identify the designs featuring the required operating frequency. On the other hand, the proposed procedure turned successful for all circuits. Table 3 provides information about the final designs, as well as the actual operating conditions therein.

**Table 3:** Optimization results for Circuits I through IV.

Circuit	Target operating parameters		Actual operating parameters		Geometry parameter values	Computational cost
	Center frequency* f*_0_ [GHz]	Power split ratio *K *[dB]	Center frequency* f*_0_ [GHz]	Power split ratio *K *[dB]		
I	1.0	0.0	1.0	0.15	x* = [0.72 0.62 11.7 15.9 1.37 0.68 0.41 0.35 4.53 0.60]^*T*^	104
II	1.0	0.0	1.0	0.1	x* = [5.53 14.4 22.0 1.07 0.90 0.85]^*T*^	130
III	2.0	0.0	2.0	0.0	x* = [1.10 1.01 0.74 0.11 1.23 0.35 6.09 2.02]^*T*^	61
IV	1.2	3.0	1.2	3.0	x* = [21.7 0.53 0.69 3.86 0.64 23.0 0.22 0.45 0.56 0.55 0.52 0.33]^*T*^	135

	Case study					
	Circuit I	Circuit II	Circuit III	Circuit IV		
Substrate	AD300 (*ε*_*r*_ = 2.97, *h* = 0.76 mm)	RO4003 (*ε*_*r*_ = 3.38, *h* = 0.762 mm)	FR4 (*ε*_*r*_ = 4.4, *h* = 1.0 mm)	FR4 (*ε*_*r*_ = 4.4, *h* = 1.0 mm)		
Design parameters	***x*** = [*g l*_1*r*_* l*_*a*_* l*_*b*_* w*_1_ *w*_2*r*_* w*_3*r*_* w*_4*r*_* w*_*a*_* w*_*b*_]^*T*^	***x*** = [*l*_1_* l*_2_* l*_3_* d w w*_1_]^*T*^	***x*** = [*G g*_1_ *g*_2_ *g*_3_ *w*_1_ *w*_3_ *L*_1_ *L*_2_]^*T*^	***x*** = [*W w*_1_*r*_* w*_2_*r*_* w*_3_ *w*_4_ *L L*_1_*r*_* L*_2_*r*_* L*_3_ *L*_4_ *L*_5_*r*_* s*]^*T*^		
Other parameters	*L* = 2*dL* + *L*_*s*_, *L*_*s*_ = 4*w*_1_ + 4* g* + s + *l*_*a*_ + *l*_*b*_, *W* = 2*dL* + *W*_*s*_, *W*_*s*_ = 4*w*_1_ + 4* g* + *s* + 2*w*_*a*_, *l*_1_ = *l*_*b*_*l*_1*r*_, w_2_ = *w*_*a*_*w*_2*r*_, *w*_3_ = *w*_3*r*_*w*_*a*_, and *w*_4_ = *w*_4*r*_*w*_*a*_, *w*_*c*_ = 1.9 mm	*d*_1_ = *d* +|*w* – *w*_1_|, *d* = 1.0, *w*_0_ = 1.7, and *l*_0_ = 15 mm	*L* = 4*w*_1_ + 10*w*_3_ + 15*g*_3_ + 2*L*_2_, *W* = 4*w*_3_ + 2*L*_1_ + *G* + 2*g*_1_ + 2*g*_3_	-		
Target operating frequency	*f*_0_ = 1 GHz	*f*_0_ = 1 GHz	*f*_0_ = 2 GHz	*f*_0_ = 1.2 GHz		
Target power division ratio	*K* = 0 dB	*K* = 0 dB	*K* = 0 dB	*K* = 3 dB		
Initial design	***x***^(0)^ = [0.5 0.52 7.0 8.5 0.5 0.4 0.15 0.15 2.0 0.3]^*T*^	***x***^(0)^ = [5.3 13.3 21.5 0.96 0.89 0.90]^*T*^	***x***^(0)^ = [1.0 1.0 0.6 0.25 2.4 0.25 9.0 3.75]^*T*^	***x***^(0)^ = [12.0 0.5 0.8 3.0 2.4 12.0 0.4 0.6 1.2 1.0 0.8 0.51]^*T*^		
Operating parameter at ***x***^(0)^	*f*_*a.*0_ = 2.2 GHz *K* = 1.2 dB	*f*_*a.*0_ = 2.1 GHz *K* = 0.2 dB	*f*_*a.*0_ = 1.05 GHz *K* = 1 dB	*f*_*a.*0_ = 2.7 GHz *K* = 3 dB		

It can be observed that both the operational frequency as well as power split ratio are well aligned with the targets. The misalignment of the center frequency is less than one percent, whereas the power division error is below or equal to 0.15 dB (it is much lower for Circuits III and IV). It should also be emphasized that the latter can be further reduced by increasing the penalty coefficient associated with the power-split-related penalty function (at the expense of slightly misaligned center frequency). At the same time, the circuit matching and isolation is also well controlled (typically, |*S*_11_| and |*S*_41_| are well below –20 dB at the target frequency *F*_*t*.0_). Finally, computational efficiency of our algorithm is excellent. The cost of the optimization process corresponds to only 104, 130, 61, and 135 EM simulations of Circuits I through IV, respectively (the average of 107). This corresponds to the typical cost of local gradient-based tuning. These expenses are low given significant relocation of the designs, normally achievable with the use of global search procedures.

A comment should be made concerning the scalability of the presented method. Based on the presented evidence of four test cases of the dimensionality ten, six, nine, and twelve parameters, as well as the cost of design optimization procedure (104, 130, 61 and 135 EM analyses per design), it might be estimated that the computational expenses are around ten times larger than the number of the design variables, which can be considered as low. In general, our procedure exhibits complexity typical for gradient-based algorithms employing first-order sensitivities, i.e., the dependence of the computational cost on the number of design variables is slightly higher than linear. In other words, the cost of the proposed procedure is comparable to that of the local optimization routines, and, as such, may be considered as practically acceptable, even for problems described by as high as twenty parameters.

**Figure 7:**
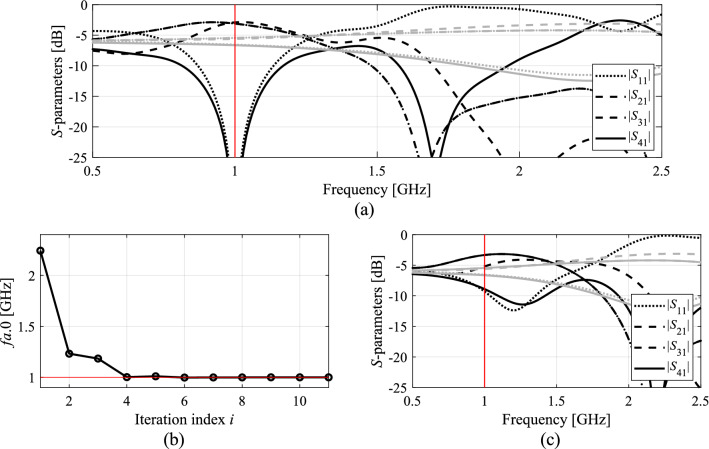
Optimization results for Circuit I: (**a**) EM-evaluated *S*-parameters at the optimized (black) and initial design (grey); (**b**) evolution of the operating frequency *f*_*a.*0_; (**c**) *S*-parameters upon first concurrent scaling (black) versus responses at the initial design (grey).

**Figure 8:**
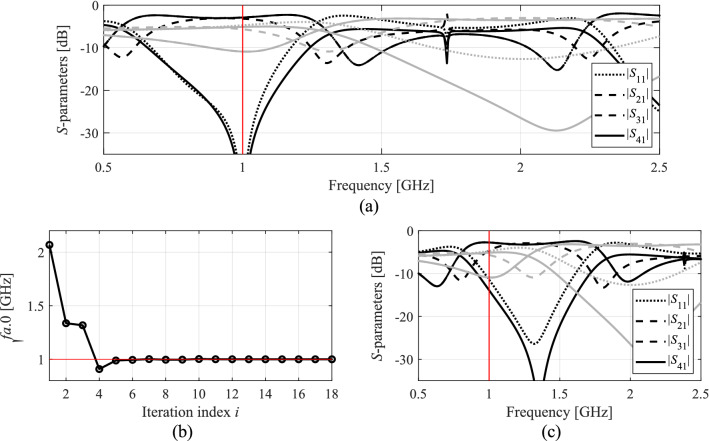
Optimization results for Circuit II: (**a**) EM-evaluated *S*-parameters at the optimized (black) and initial design (grey); (**b**) evolution of the operating frequency *f*_*a.*0_; (**c**) *S*-parameters upon first concurrent scaling (black) versus responses at the initial design (grey).

**Figure 9:**
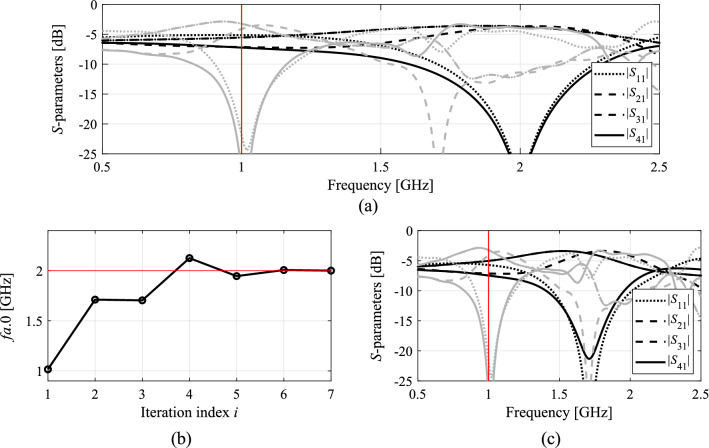
Optimization results for Circuit III: (**a**) EM-evaluated *S*-parameters at the optimized (black) and initial design (grey); (**b**) evolution of the operating frequency *f*_*a.*0_; (**c**) *S*-parameters upon first concurrent scaling (black) versus responses at the initial design (grey).

**Figure 10:**
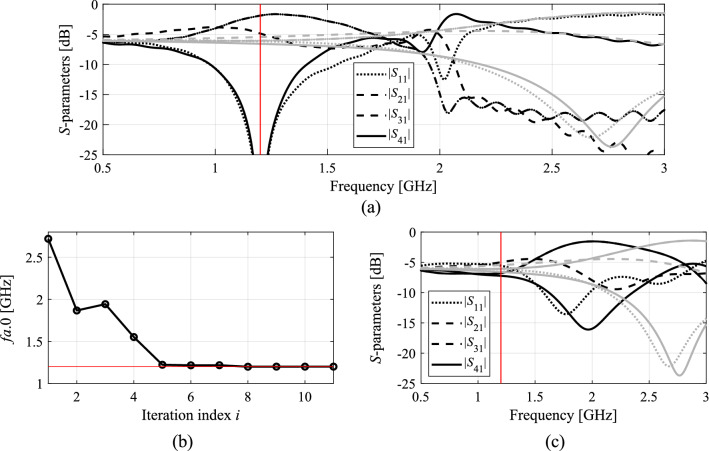
Optimization results for Circuit IV: (**a**) EM-evaluated *S*-parameters at the optimized (black) and initial design (grey); (**b**) evolution of the operating frequency *f*_*a.*0_; (**c**) *S*-parameters upon first concurrent scaling (black) versus responses at the initial design (grey).

Figures 7, 8, 9 and 10 show the circuit responses at the initial and final designs, as well as the responses at the designs obtained by the first round of concurrent scaling, along with the evolution of the current operational frequency of the coupler throughout the optimization run. It should be emphasized that despite large discrepancies between the operating frequency at the initial designs and the targets, the proposed algorithm ensures good alignment of *f*_*a.*0_ and *F*_*t.*0_ after just five iterations. The remaining computational budget is utilized to improve the objective function value at *F*_*t*.0_. Finally, it should also be mentioned that the results are consistent for all considered circuits. On the one hand, this demonstrates the overall efficacy of the presented framework. On the other hand, it shows that problem-specific tuning of the control parameters (cf. Table 2) is not necessary.

The procedure introduced in this work has been developed for handling single-band structures. Its generalization for multi-band circuits poses certain challenges, because concurrent parameter scaling as described in Sect. "Concurrent parameter scaling" would be inadequate here, due to synchronized relocation of the operating bands it would incur. The aim of the forthcoming work will be to generalize this stage of the process to allow “orthogonal” scaling of geometry variables in order to enable independent control over several center frequencies.

## Conclusion

The paper outlined a novel technique for reliable re-design of microwave passives over broad ranges of operating frequencies. Our methodology utilizes a scope-controlled concurrent scaling of geometry parameters interleaved with local tuning. The former enables large design relocations at minimum computational expenses, whereas the latter allows for a continuous improvement of the design with regard to the assumed performance figures. Both mechanisms have been incorporated into an optimization framework controlled by a few user-defined and easily adjustable parameters. Comprehensive verification involving four compact microstip couplers demonstrated superior efficacy of the procedure, both in terms of reliability, and computational efficiency. In particular, it permits a precise control of the center frequencies and other electrical performance figures (here, a power division ratio), while being able to re-design the structures to operating frequencies distant from those at the initial designs. The cost of the optimization procedure slightly exceeds one hundred EM evaluations of the device under design, thus, it is equivalent to the expenses associated with a local gradient-based search. At the same time, straightforward local optimization failed for all test circuits, which indicates, that—for the considered design scenarios—global search routines would normally be necessary.

The presented technique has been developed to handle single-band circuits, which is a practical limitation as contemporary applications often require dual-, triple-, or even quad-band structures. Generalization of the method for multi-band systems poses considerable challenges. In particular, simple concurrent parameter scaling results in more-or-less synchronized adjustment of the operating bands. Enabling independent control requires more sophisticated strategies, involving a number of scaling directions, which are “orthogonal” to each other in terms of their effects on particular operating frequencies. The focus of the future work will be the conceptual development and implementation of the respective design framework, as well as its verification using real-world test cases.

## Data Availability

The datasets generated during and/or analysed during the current study are available from the corresponding author on reasonable request.
